# A Hydrogel-Based Ultrasonic Backscattering Wireless Biochemical Sensing

**DOI:** 10.3389/fbioe.2020.596370

**Published:** 2020-11-27

**Authors:** Juhong Nam, Eunjeong Byun, Hyunji Shim, Esther Kim, Sayemul Islam, Moonchul Park, Albert Kim, Seung Hyun Song

**Affiliations:** ^1^Department of Electronics Engineering, Sookmyung Women's University, Seoul, South Korea; ^2^Department of Electrical and Computer Engineering, Temple University, Philadelphia, PA, United States

**Keywords:** biochemical sensing, ultrasonic, implantable sensor devices, hydrogel, wireless sensing

## Abstract

Wireless monitoring of the physio-biochemical information is becoming increasingly important for healthcare. In this work, we present a proof-of-concept hydrogel-based wireless biochemical sensing scheme utilizing ultrasound. The sensing system utilizes silica-nanoparticle embedded hydrogel deposited on a thin glass substrate, which presents two prominent interfaces for ultrasonic backscattering (tissue/glass and hydrogel/glass). To overcome the effect of the varying acoustic properties of the intervening biological tissues between the sensor and the external transducer, we implemented a differential mode of ultrasonic back-scattering. Here, we demonstrate a wireless pH measurement with a resolution of 0.2 pH level change and a wireless sensing range around 10 cm in a water tank.

## 1. Introduction

Assessments of the analytes in biofluids through biochemical monitoring have been a major goal for healthcare. To achieve this, much progresses have been made in developing different biochemical monitoring modalities lab-on-a-chip, wearable sensors, implantable sensors, and etc. (Cheng and Chau, 2003; Samiei et al., 2016; Kim et al., 2017; Heikenfeld et al., 2018, 2019). Among them, although invasive, implantable sensors are perhaps the only modality that provides direct access to the biofluids and hence greater signal-to-noise ratios (Darwish and Hassanien, 2011; Heikenfeld et al., 2019).

Hydrogels, crosslinked polymer networks that absorb a large amount of water, are particularly suitable for implantable sensors as they do not require external power sources and exhibit reversible volume and shape response to a variety of chemical stimuli such as pH, ions (Xu et al., 2007), antigens (Miyata et al., 1999), temperature (Zhang and Zhuo, 2000; Liu and Fan, 2002), mechanical stimuli (Liu et al., 2012; Huebsch et al., 2014), and glucose (Hassan et al., 1997; Lee et al., 2004; Siegel et al., 2010). In addition, the swelling/deswelling responses of hydrogels typically do not involve the use of enzymes or catalysts; thus, the lifetime of the hydrogel based systems can be much longer than those involving enzymatic reactions (Hassan et al., 1997; Heo et al., 2011). For these advantages, numerous hydrogel-based systems with many different applications including sensors, electronics, and soft robotics have been reported (Hassan et al., 1997; Qiu and Park, 2001; Lee et al., 2004; Lei et al., 2006; Heo et al., 2011; Song et al., 2014; Cangialosi et al., 2017; Park et al., 2018; Yang and Suo, 2018; Yuk et al., 2019).

In the wireless sensor applications, the volume response of a hydrogel should first be transduced to a measurable quantity such as voltage, capacitance, inductance, and etc. (Hassan et al., 1997; Lee et al., 2004; Lei et al., 2006; Heo et al., 2011; Song et al., 2014; Park et al., 2018). While RF electronics is standard for the wireless interrogation, its complexity is not suitable for the hydrogel-based sensor. In contrast, ultrasound can be coupled with the hydrogel's volume response to infer the target stimuli. The ultrasound can offer unique advantages including a long interrogation depth and small sensor dimensions, and simplicity as demonstrated by some of the previous works (Tro¨ıani et al., 2011; Park et al., 2018; Farhoudi et al., 2020). In order to achieve the high accuracy, however, the ultrasound interrogation scheme should account for the scattering in biological tissues, which are often unpredictable in its nature (Shung and Thieme, 1992). The variation is attributed to the continuously changing acoustic properties of the biological tissues due to different conditions over time (e.g., hydration, movements, etc.), resulting in distorted echos between an ultrasonic probe and the target (Shung and Thieme, 1992).

**Figure 1:**
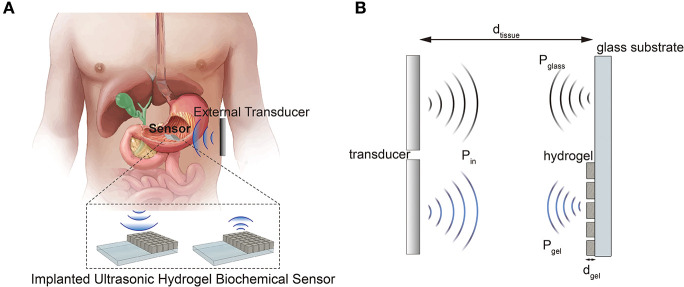
**(A)** Schematic illustration of a proposed sensor system application. **(B)** Operation principle of the differential ultrasonic biochemical sensor.

In this paper, we demonstrated an ultrasonic hydrogel biochemical sensor system operated in a pulse-echo mode using a prototype pH-sensitive hydrogel sensor. The hydrogel is also featured with micro-patterning to achieve isotropic swelling/deswelling. We believe the proposed sensing scheme can potentially be utilized as an implantable wireless biochemical sensor (Figure 1A). The sensor operates in a differential mode in which the two acoustic waves travel through two different paths; one with and without the silica-loaded hydrogel in the path (Figure 1B). The additional attenuation due to the hydrogel, especially by embedded silica nanoparticles, changes as a function of hydrogel's volume. Thus, the normalized ratio of ultrasonic properties (i.e., attenuation) between the two interfaces (hydrogel/glass and tissue/glass) is independent of the varying tissue properties (e.g., hydration, movements, etc.) and only depends on the hydrogel's volume response to the target biochemical stimuli.

## 2. Materials and Methods

### 2.1. Reagents and Materials

3-(trimethoxysilyl)propyl methacrylate (γ -MPS), acrylamide(AAm), N,N′-methylenebisacrylamide(BIS), methacrylic acid(mAA), N,N,N′,N′-tetramethylethylenediamine(TMEDA) were purchased from Sigma-Aldrich (USA). Ammonium persulfate(APS) and standard buffer solutions were purchased from Daejung Chemicals & Metals (South Korea). Silica dioxide nanoparticles (SiO_2_, 99+%, 400 nm) were purchased from U.S. Research Nanomaterials (USA). PZT plates were purchased from Mide Technology (USA).

### 2.2. Synthesis of Silica-Loaded Hydrogel

The silica-loaded pH-sensitive hydrogel was synthesized by mixing two pre-gel solutions A and B in a 6:1 ratio; the hydrogel synthesis recipe is based on previous reports (Lei et al., 2006; Ding et al., 2010). The pre-gel solution A was prepared by mixing 280 mg of acrylamide (AAm, Sigma-Aldrich), 84 μL of methacrylic acid (mAA, Sigma-Aldrich), 83 μL of N,N,N',N'-tetramethylethylenediamine (accelerator, TMEDA, Sigma-Aldrich), and 13.6 mg of N,N'-methylenebisacrylamide (crosslinker, Bis, Sigma-Aldrich) per 1 mL of DI water. The solution A was stirred in at room temperature for 3 h for the complete dissolution of the chemicals followed by the addition of the silica nanoparticles. The concentration of the silica particles were determined by the weight/volume percentage (g/ml) of the total pre-gel solutions. The pre-gel solution B was prepared by dissolving 80 mg/mL ammonium persulfate (initiator, APS, Sigma-Aldrich) in DI water. The amount of accelerator was tuned for a rapid polymerization (within 1 min) to minimize the capillary flow and the settling of silica nanoparticles. For the hydrogel used in our prototype device, the carboxyl groups of the methacrylic acid ionize at alkaline pH; the electrostatic repulsion between the ionized carboxyl groups results in increased hydrogel volume (Lei et al., 2006; Pérez-Álvarez et al., 2008; Ding et al., 2010; Song et al., 2014; Park et al., 2018).

### 2.3. Characterization of the Swelling Ratio of the Silica-Loaded Hydrogel

Although the swelling/deswelling behaviors of the pH-sensitive mAA-co-AAm hydrogel itself were well-studied in our previous investigations (Pérez-Álvarez et al., 2008; Ding et al., 2010; Song et al., 2014; Park et al., 2018), the effect of silica nanoparticle loading on such hydrogel needs to be characterized. To study the effect of the silica nanoparticle loading on the pH-gel swelling behavior, we firstly fabricated hydrogel cuboids (5 × 5 × 3*mm*^3^) with three different silica loading concentrations of 0, 5, and 10 w/v% with a 3D printed acrylic mold (Form2, Formlabs, USA). The mixed pre-gel solutions were poured into the mold. After the complete polymerization of the hydrogel (≈ 10 min), the cuboids were removed from the mold. The silica-loaded hydrogel cuboids were then immersed in buffer solutions with different pH, ranging from 3 to 9, for 24 h. The swelling behaviors of the silica-loaded hydrogel were characterized by a stereo microscope (Leica MZ10F).

### 2.4. Device Fabrication

**Figure 2:**
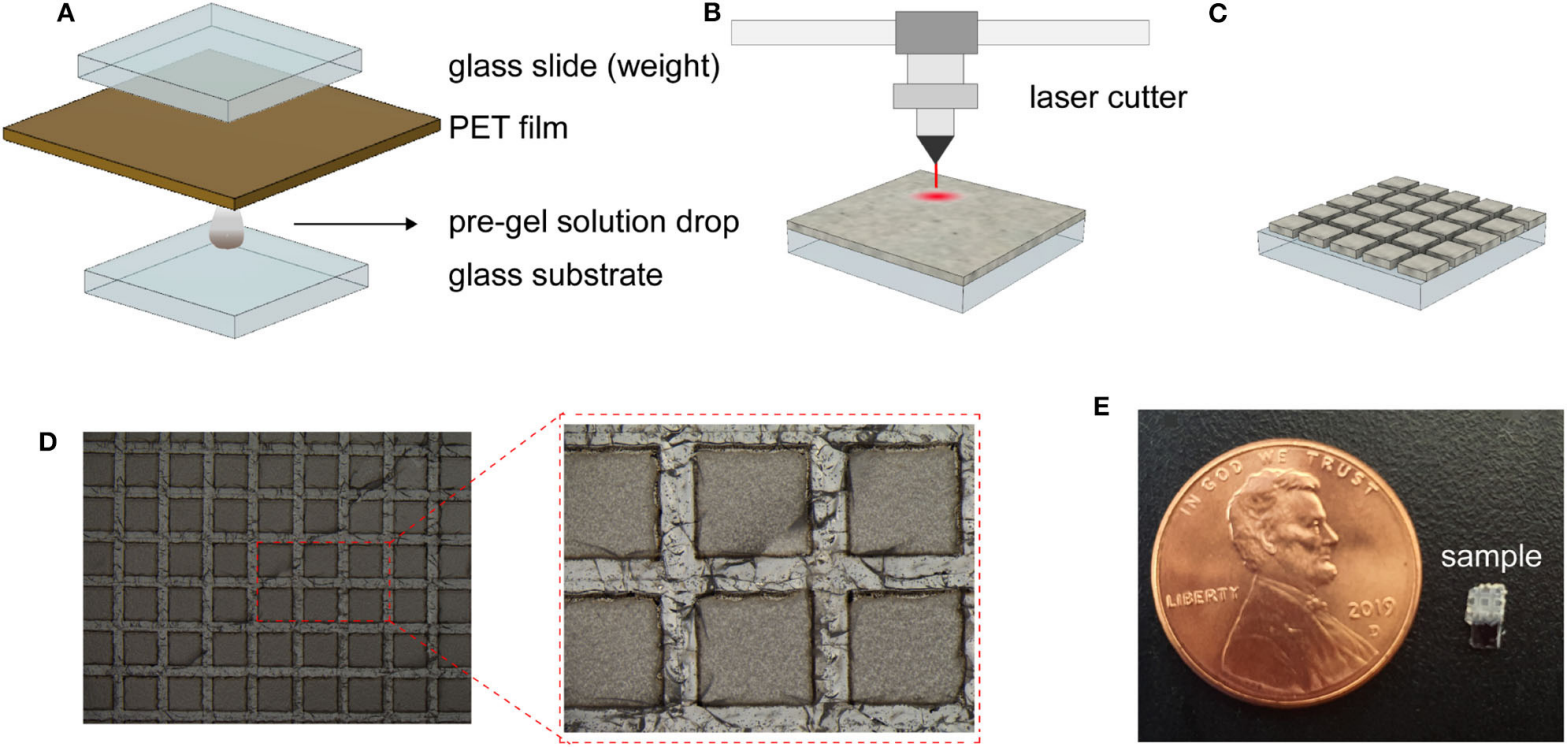
Sensor fabrication steps and resulting sensor. **(A–C)** Schematic illustration of the sensor fabrication of the hydrogel deposition **(A)**, laser patterning of the dehydrated hydrogel **(B)**, and the resulting patterned hydrogel surface **(C)**. Optical photograph of the fabricated dehydrated hydrogel **(D)**.Photograph of the prototype device with a overall dimension of 2 × 4 × 0.5 mm **(E)**.

To promote the adhesion between the substrate and the hydrogel, the substrates (glass slides and oxidized silicon chip) were firstly pre-treated in a 10 vol % solution (in acetone) of organosilane coupling agent (γ -MPS) for 1 h, rinsed with IPA and acetone and dried in an oven(120 ^*o*^C) for 10 min. A silica loaded hydrogel pre-gel solution (15 w/v%) was deposited on the pre-treated substrate (Figure 2A) by squeeze film casting (Ding et al., 2010). The resulting sample of silica-loaded hydrogel on glass substrate was completely dehydrated. The hydrogel film was then cut into squares of (500 μm^2^) with 200 μm spacing using a CO_2_ laser cutter (C1280-150W, e-laser) (Figures 2B,C). The patterns (shown in Figure 2D) were introduced to improve response time and reversibility by reducing the strain build-up in the gel (Song et al., 2014). The patterned hydrogel sensor was then immersed in DI water to extract the burnt polymers and unreacted monomers overnight. The final prototype device (dimensions of 2 × 4 × 0.5 mm) is shown in Figure 2E.

### 2.5. Measuring Ultrasonic Intensity and Waveform Characterization Using Hydrophone

The acoustic intensity of the applied ultrasound was measured by a fiber optics hydrophone (Precision Acoustics, UK) connected to an oscilloscope (MDO3054, Tektronix). The hydrophone was placed at the focal plane of the transducer. The pressure of the applied ultrasound was obtained by dividing the voltage output of the hydrophone (*V*_*hp*_) by the hydrophone's sensitivity (*S*_*hp*_).

(1) $$\begin{array}{l} p=\frac{V_{hp}}{S_{hp}}\;\left[MPa\right] \end{array}$$

The peak pressure was then converted to the average acoustic intensity by the following equation (I: acoustic intensity [W/m^2^], p: peak pressure [Pa], ρ: water density [kg/m^3^], c: sound velocity in water [m/s]).

(2) $$\begin{array}{l} I=\frac{p^{2}}{2\rho \cdot c}\;\left[W/m^{2}\right] \end{array}$$

### 2.6. Measurement Setup for Ultrasonic pH Sensing

**Figure 3:**
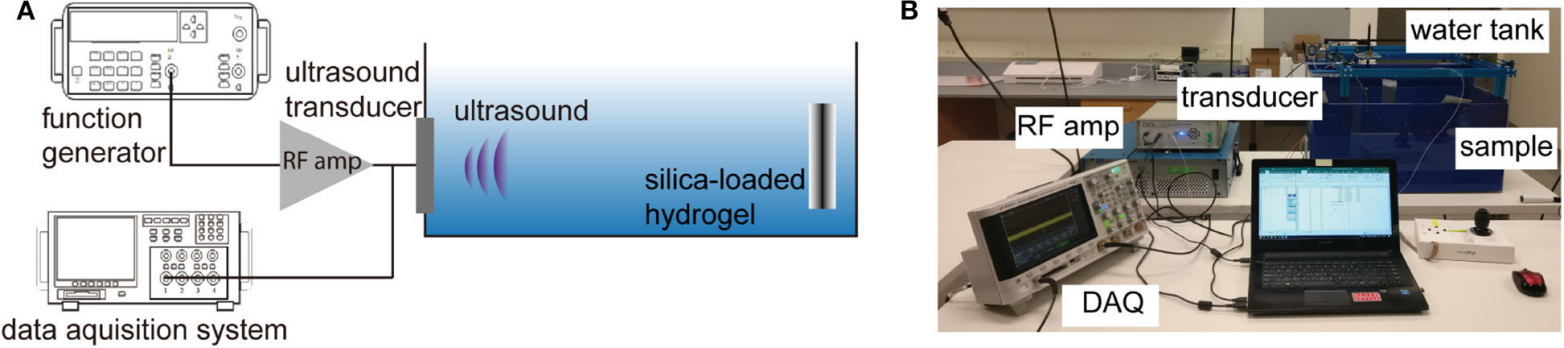
**(A)** Schematic depiction of the ultrasonic measurement of pH sensor, **(B)** representative photo of the experimental setup.

The experimental setup for the wireless ultrasonic pH sensing is shown in Figures 3A,B. The hydrogel sensor and the transducer were immobilized by a custom 3D printed holder; the distance between the transducer and the sensor was fixed at 8.5 cm. The water tank is used due to the similarity in acoustic properties to soft tissues. The custom ultrasonic transducer was driven at its resonant frequency using a function generator (Tektronix AFG1022) connected to an RF amplifier (E & I 240L) at a pulse mode (input sinusoidal: 2.3 MHz, wavelength = 0.6 mm, input V_*pp*_ = 500 mV, number of pulse = 1, interval = 5 ms). The ultrasonic waves reflected at the sensor interface were captured by the transmitting transducer connected to an oscilloscope.

## 3. Results

### 3.1. Characterization of Free Swelling of Bulk Hydrogel With Silica Nanoparticles

**Figure 4:**
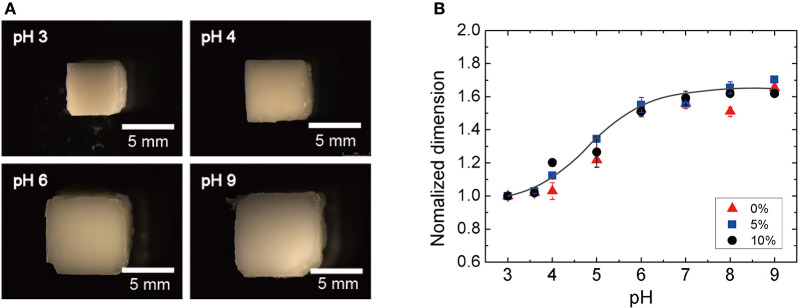
Swelling ratio as a function of silica-loading concentration: **(A)** optical photographs of the silica-loaded cuboid at different pH and **(B)** normalized dimension of the hydrogel as a function of pH.

The silica nanoparticle loading is introduced to the hydrogel network to increase the scattering of the propagating ultrasonic waves, which enables the inference of the hydrogel volume from the pressure ratio of the backscattered ultrasonic waves (Equation 5). However, high loading concentrations of the silica nanoparticles can potentially retard the swelling behavior of the hydrogel. Thus, we investigated how the pH-sensitive hydrogel swelling behaviors are affected by the particle concentration. The identical silica-loaded hydrogel cuboids were immersed in buffer solutions with different pH levels ranging from 3 to 9 for 24 h prior to characterization. Figure 4A show the optical microscope images of hydrogel cuboids (10 w/v %) equilibrated at four different pH levels of 3, 4, 6, and 9. The normalized dimensions of the hydrogels at different pH (normalized by the dimension at pH 3) are summarized in Figure 4B. The observed hydrogel swelling behaviors were consistent with the previous reports with a sharp volume change between pH of 3 to 6 (Lei et al., 2006; Quesada-Pérez et al., 2011). Moreover, the swelling behaviors remained unaffected by the embedded silica loading up to 10 w/v% with 4-fold (1.6^3^) maximum volume increase.

### 3.2. Characterizing the pH-Sensitive Ultrasonic Hydrogel Sensor

**Figure 5:**
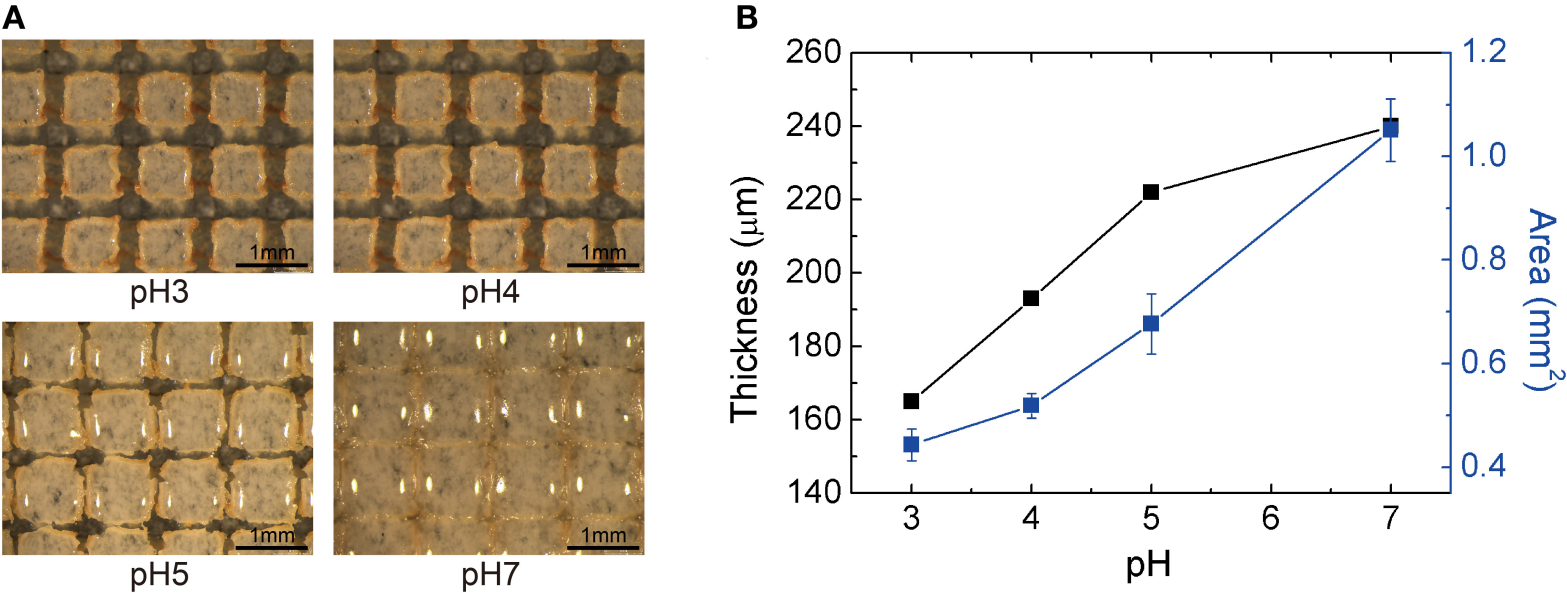
Volumetric response of silica-loaded hydrogel sensor **(A)** optical photographs of the patterned hydrogel at different pH levels (scale bar length = 1 mm) **(B)** the thickness (μm) and the area per block (mm^2^) as a function of pH.

Applying the silica-loaded hydrogel to the sensor geometry (i.e., thin film; thickness ≈ 100 μm) requires adhesion between the hydrogel and the substrate. In our fabrication, the organosilane coupling agents covalently bond the hydrogel to the substrate; this would result in a different swelling behavior of the hydrogel since one interface is clamped. To maintain the equal swelling behavior as a function of pH, we micropatterned the hydrogel to create cubical form factor (i.e., equal in width, length, and thickness). Figure 5A shows the photos of the hydrogel (optimized at 100 μm thick when dehydrated) under different pH levels. The thickness and the surface area of an individual block of silica-loaded hydrogel pattern is shown in Figure 5B. The normalized swelling ratio in the thickness direction with one interface clamped was 1.5, similar to that of free swelling of bulk hydrogel (1.6). Accompanied by the increase in thickness, the lateral dimensions of the hydrogel expand: at pH 7, the blocks almost come into contact with the neighboring blocks. Thus, we conclude that introducing patterning is critical for preventing the hydrogel blocks can delaminate from the substrate due to the excess internal stress buildup and ensuring the reversible swelling behavior of the hydrogel.

With the confirmed volume response of the silica-loaded hydrogel sensor, we carried out the ultrasonic pH measurement with the prototype device. Figure 3A shows the schematic illustration of the experimental setup, where the hydrogel's volume response is interrogated using the proposed differential pulse-echo measurement scheme. We chose differential measurement scheme that normalizes the reflection from the gel/substrate interface against the water/substrate interface, which is determined only by the attenuation due to the silica-embedded hydrogel. The received reflected ultrasonic wave from the gel/glass and water/glass interfaces can be expressed as below.

(3) $$\begin{array}{l} P_{gel/glass}=\frac{A_{gel}}{A_{sample}}\cdot P_{0}e^{-2\alpha_{tissue}d_{tissue}}e^{-2\alpha_{gel}d_{gel}}\\ \;+\frac{A_{sample}-A_{gel}}{A_{sample}}\cdot P_{0}e^{-2\alpha_{tissue}d_{tissue}} \end{array}$$

(4) $$\begin{array}{l} P_{water/glass}=P_{0}e^{-2\alpha_{tissue}d_{tissue}} \end{array}$$

*P*_0_ is the initial acoustic pressure of the transmitted ultrasonic wave from the transducer, *A*_*gel*_ is the area of the hydrogel, *A*_*sample*_ is the area of the sample surface interacting with the ultrasound, *d*_*tissue*_ is the distance between the transducer and the sample surface, α_*tissue*_ is the attenuation coefficient of the tissue, *d*_*gel*_ is the thickness of the hydrogel, and α_*gel*_ is the attenuation coefficient of the silica-loaded hydrogel. While the varying tissue properties (e.g., hydration, movements, etc.) can affect the attenuation of the ultrasonic waves in the biological tissues, the normalized ratio between the two measured pressures is independent of the tissue properties (Equation 5).

(5) $$\begin{array}{l} attenuation\;ratio=\frac{P_{gel/glass}}{P_{water/glass}}\\ \;=\frac{A_{gel}}{A_{sample}}e^{-2\alpha_{gel}d_{gel}}+\frac{A_{sample}-A_{gel}}{A_{sample}} \end{array}$$

Since the area, the thickness, and the attenuation coefficient of the hydrogel are functions of the hydrogel's volume, the target analytes can be inferred from the simple backscattered ultrasonic waves. We note that the Equations (3)–(5) were derived based on the ultrasonic transmission, similar to formulations presented in the pulse-echo ultrasonic fingerprint sensors (Lu et al., 2015; Tang et al., 2015).

**Figure 6:**
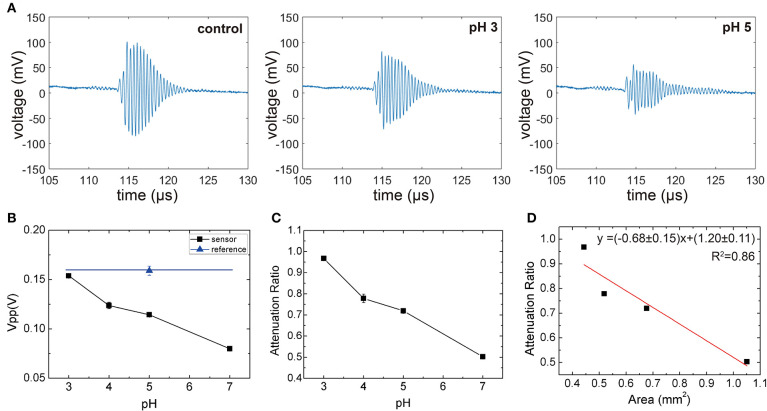
**(A)** The representative waveform of control and sensor (pH 3 and pH 5), **(B)** peak voltage output of the transducer for the reference and sensor at different pH levels, **(C)** ratio between the sensor output and reference output as a function of pH. All measurements were repeated five times to obtain the standard error. **(D)** Attenuation ratio as a function of gel area. The slope fitted via Equation (5) (red line) is approximated to -(1-c).

The sensor responses as a function of pH are summarized in Figure 6. As expected, the amplitudes of the reflected ultrasound of the reference interface [i.e., without silica-loaded hydrogel (blue)] were independent of pH. On the other hand, the sensor output demonstrates a significant pH dependence compared to the reference. The attenuation due to the hydrogel becomes more pronounced at higher pH. Taking the ratio between the reference and the sensor output allows the output to be independent of the intermediate medium (Equation 5). Figure 6A shows the pH response of the sensor (Confidence interval for each point of Figure 6C is 0.97±0.021, 0.78±0.086, 0.72±0.048, 0.50±0.016).

To rationalize the observed monotonic decrease, which is a result of competing effects of decreased attenuation coefficient due to lower silica concentration and the increased hydrogel thickness and area, we plotted the attenuation coefficient as a function of hydrogel area per block (Figure 6D). We observed that The attenuation coefficient as a function of hydrogel area per block showed a linear trend, which implies that the attenuation through the silica-loaded hydrogel is relatively constant (i.e., $e^{-2\alpha_{gel}d_{gel}}\approx c$). By applying this approximation to Equation (5), we obtain Equation (6), which shows that the fractional area of the hydogel (*A*_*gel*_/*A*_*sample*_) will determine the attenuation ratio. Thus, we will expect a linear relationship between the areal coverage of the hydrogel and the attenuation ratio as shown in Figure 6D. We note that although the attenuation through the silica-loaded hydrogel is not a constant throughout different pH levels, the Equation (6) describes the attenuation ratio as a function of the surface area reasonably well as a first approximation. This in turn illustrates the importance of the silica inclusion since the sensitivity, roughly denoted as $1-e^{-2\alpha_{gel}d_{gel}}$, will be higher for the greater attenuation through the hydrogel). The sensor resolution, estimated from the standard errors is about 0.2 pH unit.

(6) $$\begin{array}{l} attenuation\;ratio\approx 1-\left(1-c\right)\times \left(\frac{A_{gel}}{A_{sample}}\right) \end{array}$$

**Figure 7:**
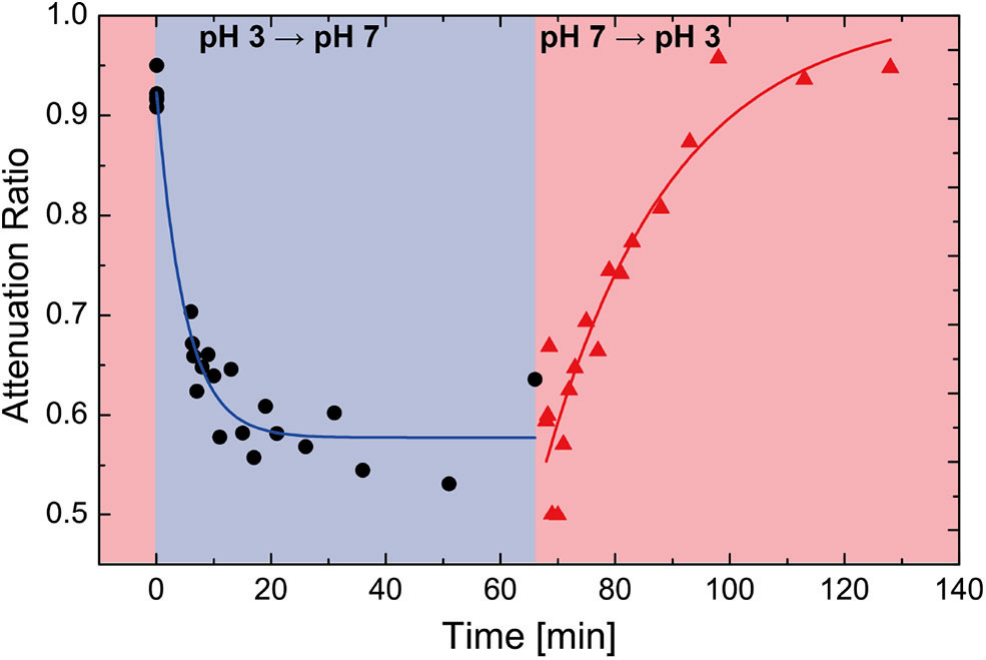
Time evolution data of the ultrasonic hydrogel pH sensor. The shaded background indicates the pH of the buffer solution that the sensor is embedded in red: pH 3, blue: pH 7.

Lastly, we confirmed the reversible sensing capability of the ultrasonic hydrogel sensor. Prior to the experiment, the silica-loaded hydrogel sensor was immersed in pH 3 media until a stable ultrasonic reading was obtained (~ 1 h). At *t* = 0, the media was replaced with pH 7 buffer (the complete solution exchange occurred within a minute). Immediately following the pH change from 3 to 7 (blue shaded area of Figure 7), the sensor output changed and stabilized around 0.55, which was consistent with the prior sensor characterization (Figures 6B,C). The dynamics of the sensor was well-described with an exponential decay (blue line) with a time constant of 4.9 min. A reversible sensing capability was observed when the pH was changed back to 3 (the red shaded area). The sensor also exhibited the exponential decay behavior with a time constant of 22.5 min. The smaller time constant of the swelling is attributed to different driving mechanisms of the swelling/deswelling behaviors of pH sensitive hydrogel; the swelling is driven by the electrostatic repulsion between the deprotonated carboxyl groups while the deswelling is governed by the diffusion of water diffusion, which tends to be slower than the former (Katime et al., 1999). Also, the final sensor output stabilized around 0.95, recovering to its initial value at *t* = 0.

## 4. Discussion

The proof-of-concept ultrasonic hydrogel pH sensor presented in our work successfully measured pH with a working distance of ≈ 10 cm using moderately weak acoustic intensity (<200 mW/cm^2^). The sensing distance could easily be extended with the use of a higher ultrasonic intensity as long as it is under the FDA regulation on the imaging ultrasonic intensity (720 mW/cm^2^). The proposed sensing modality using a differential pulse-echo scheme is enabled by the sensor geometry of patterned gel and a reference surface. The introduction of the silica-nanoparticles serves as the scattering medium for the propagating ultrasound. Moreover, the patterning of the hydrogel enables reversible response of the hydrogel swelling behaviors.

Despite these encouraging results, we recognize that the further studies and developments are necessary for the clinical application of the device and the readout system. Firstly, further reduction of the sensor dimension will be required for a realistic application of the sensor system especially in the thickness. The decrease in the thickness will also speed up the response time, typically determined by the diffusion of the analytes, scales with the square of the thickness. In addition, the effect of the misalignment must be addressed and compensated. Moreover, the integrated external transceiver system that combines ultrasonic transducer and signal processing would be necessary for the continuous monitoring of the biochemical analytes. Lastly, the sensor will need to be characterized in a restricted system that can more precisely mimic the body tissue implants.

Nevertheless, the presented results clearly indicate that (1) our prototype device can perform biochemical sensing based on backscattered ultrasonic waves and (2) the general method presented in our work can be easily adopted to different analytes by incorporating other types of hydrogel, which is sensitive to different analytes. Thus, our hydrogel-based ultrasonic backscattering wireless biochemical sensing scheme has a potential to be a long-term implantable biochemical sensing solution.

## Data Availability Statement

The raw data supporting the conclusions of this article will be made available by the authors, without undue reservation.

## Author Contributions

SS conceived the idea. Under the guidance of SS, JN, EB, HS, and EK carried out the experiments and analyzed the data. Under the guidance of AK, SI, and MP examined initial feasibility. JN, AK, and SS wrote the manuscript. All authors read and commented on the manuscript.

## Conflict of Interest

The authors declare that the research was conducted in the absence of any commercial or financial relationships that could be construed as a potential conflict of interest.
